# Survey of lived experiences and challenges in hepatitis B management and treatment

**DOI:** 10.1186/s12889-024-18425-w

**Published:** 2024-04-02

**Authors:** Catherine Freeland, Charles Adjei, Jack Wallace, Su Wang, Jessica Hicks, Danjuma Adda, Cary James, Chari Cohen

**Affiliations:** 1 Hepatitis B Foundation, Doylestown, PA USA; 2 University of Ghana, Accra, Ghana; 3 Burnet Institute, Melbourne, Australia; 4 Centre for Social Research in Health, University of New South Wales, Sydney, Australia; 5 Saint Barnabas Medical Center, Livingston, NJ USA; 6 World Hepatitis Alliance, London, UK; 7 CFID Taraba, Taraba, Nigeria

**Keywords:** Hepatitis B, Viral hepatitis, Patient outcomes, Treatment preferences

## Abstract

Almost 300 million people are living with chronic hepatitis B infection worldwide and most remain undiagnosed and at risk for liver cancer. In 2015 the World Health Organization (WHO) developed guidelines for the prevention, care, and treatment of persons with chronic hepatitis B and in early 2023 began to work on updating these guidelines. In March 2023, a self-administered, anonymous online survey was launched, aiming to identify patient preferences related to the clinical management of hepatitis B including current management, treatment, and care experiences, preferences regarding engagement with providers, and preferences related to simplifying hepatitis B care access. A sample of 560 individuals living with hepatitis B (self-identified as HBsAg positive) from 76 countries completed the survey. Key findings demonstrated that less than half (49%, *N* = 268) of participants regularly visited a doctor to check the health of their liver (every 6–12 months), with 37% of participants prescribed antiviral medication by a specialist (82%, *N* = 167) or general practitioner (13%, *N* = 26). Participants reported not being actively involved in care decision making with their providers (42%, *N* = 217), with an overwhelming majority wanting to participate in hepatitis B management and treatment choices (85%, *N* = 435). Participants provided qualitative and quantitative details using open-ended responses within the survey about challenges with medication affordability and receiving care from a knowledgeable provider. Overall findings demonstrated key gaps in care, management, and treatment access related to hepatitis B: identifying these gaps can be used to identify areas for improvement along the care continuum for viral hepatitis. The survey found a need for the comprehensive simplification of clinical management and health care services related to hepatitis B. A thematic analysis of the open-ended survey responses highlighted major overarching themes including the cost and access burdens associated with hepatitis B management and treatment, and challenges in finding knowledgeable providers. Results from this mixed methods survey were used to inform the WHO hepatitis B guidelines update. Efforts should continue to explore public health approaches to address barriers and facilitators to testing, care, and treatment for people with hepatitis B to improve awareness of hepatitis B and access, care, and treatment among patients and providers.

## Introduction

Globally, it is estimated almost 300 million people are living with chronic hepatitis B infection.^1^ In 2019, the World Health Organization (WHO) estimated that 820,000 deaths worldwide were attributed to cirrhosis or liver cancer resulting from hepatitis B infection[1]. According to the WHO, between 20% and 30% of people chronically infected with hepatitis B will develop life-threatening complications, including liver cirrhosis and hepatocellular carcinoma (HCC, liver cancer)[2,3]. In 2016 the WHO established elimination goals to reduce new hepatitis B infections by 90% and hepatitis-related deaths by 65% by 2030[2]. In May 2022, the 75th World Health Assembly renewed the hepatitis B elimination goals, integrating them with HIV and sexually transmitted infections (STIs) within a focus on universal health coverage, simplified primary health care and person-centered care [4]. These goals include reducing the number of annual hepatitis-related deaths to 4 per 100,000 by 2030 and expanding treatment coverage for those diagnosed with hepatitis B by 80% [5]. The updated Global Health Sector Strategies 2022–2030 has a common vision to end AIDS and the epidemics of viral hepatitis and sexually transmitted infections by 2030 through five strategies with one being to engage empowered communities and civil society.

Despite significant mortality, only approximately 10% of people with hepatitis B are diagnosed and therefore receive limited clinical management including treatment, particularly in low and middle-income countries (LMIC)[6, 7]. Based on the current WHO guidelines all adults, adolescents, and children with chronic infection and clinical evidence of compensated or decompensated cirrhosis should be treated. Additionally, treatment is recommended for adults with chronic hepatitis B who do not have clinical evidence of cirrhosis but are aged more than 30 years and have persistently abnormal liver enzyme levels and evidence of high-level hepatitis B replication. WHO also states that continued monitoring is necessary in all persons with chronic infection particularly in those who do not meet the treatment criteria [8]. Monitoring according to the current guidelines indicates clinical assessment at least once a year for those not within treatment criteria and more frequently if there are abnormal liver enzymes or elevated virus replication [8].

The engagement of the affected community is critical to viral hepatitis elimination, and to increase access to, and uptake of hepatitis B-related clinical services, it is important to understand the impact of hepatitis B on different aspects of a person’s life and within their communities. Designing effective programs to improve access to testing and diagnosis requires a better understanding of the lived experiences and preferences of people with hepatitis B, however, currently, there is limited data describing these preferences available. While clinical management focuses primarily on reducing the morbidity and mortality associated with hepatitis B, it is the psychological and social aspects of hepatitis B that are often the barriers to patients accessing or remaining in care [9, 10]. Understanding these dimensions will better inform the provision of care, including guideline development, and support people living with hepatitis B to achieve the elimination goals more comprehensively [11].

To gain a better understanding of people with hepatitis B and inform the WHO updated hepatitis B treatment guidelines, a survey was conducted to better understand diagnostics, treatment, and management preferences and barriers to accessing care for hepatitis B. Individuals with lived experience were asked to participate in an anonymous survey to assess the trajectory of how people with hepatitis B experience and engage with the health care system for their infection from testing, diagnosis, clinical management, treatment, and including health care challenges within those settings. Participants were also asked to describe specific preferences for overall management and care related to hepatitis B. This study reports on survey findings and provides insight into the current lived experiences of people with hepatitis B and suggestions for improving care and management. The survey results can be used to inform key updates and considerations to hepatitis B clinical management and treatment recommendations within future hepatitis B guideline development.

## Methods

### Design and objectives

In March 2023, a self-administered, anonymous online survey was launched by the Hepatitis B Foundation (U.S.) to investigate patient preferences related to the clinical management of hepatitis B including current management, treatment, and care experiences, preferences regarding engagement with providers, and preferences related to simplifying hepatitis B care.

### Survey participants

Survey recruitment occurred through community and advocacy organizations’ email listservs, newsletters, social media channels, and organizations providing outreach directly to people with hepatitis B. The online survey was available between March and June 2023. Individuals were eligible to complete the survey if they self-reported being currently infected with hepatitis B (HBsAg positive). Individuals who reported not being infected were not offered the opportunity to complete the survey.

### Survey questionnaire

Survey development was informed by a previous WHO hepatitis C patient preferences survey 12 and literature review and adapted by experts both with lived experience of viral hepatitis and/or public health researchers with key hepatitis B related issues identified to inform the WHO guidelines. The survey consisted of 27 questions that were mostly multiple choice, with 12 “other” options, and three open-ended questions available to provide additional information where appropriate. The survey enabled participants to include information they felt appropriate as considerations for treatment and the experiences and preferences associated with hepatitis B management. The survey was organized into three main sections: [1] eligibility and demographic section (five questions); [2] current clinical management including treatment experiences (seven questions), and [3] preferences for treatment, management, and provider interactions (15 questions). The survey was only available in the English language due to the required timelines to inform guidelines and the complexity and costs associated with identifying other relevant languages appropriate for the survey. Individuals were eligible to participate if they self-reported being at least age 18 and living with hepatitis B. Those not self-reporting having hepatitis B being at least age 18 were excluded from the survey.

### Ethical considerations

All participants were provided with information detailing the purpose of the survey before participation. No identifying information was collected from participants. The survey was completely voluntary and anonymous. The survey was approved by the Heartland Institutional Review Board (HIRB Project No. 171120-171) and was deemed to be of minimal risk to survey participants.

### Data analysis

Questionnaire responses were analyzed using descriptive statistics within Microsoft Excel and JMP Pro v 17. Survey data was cleaned in Excel software removing all responses to people who were not living with hepatitis B (*N* = 204). For qualitative open-ended survey responses, thematic analysis was performed through the line-by-line reading of all open-ended survey responses and identifying key overarching themes. Key quotes reflecting overarching themes assessed and reported in the text. For data visualization, JMP Pro v. 17 was used to conduct descriptive analyses to identify differences in access to care and access to treatment by the WHO regions. As there were over 70 countries reported within the data, collected under the country, this variable was collapsed into groups based on WHO defined regions of Africa, Americas, South East Asia, Mediterranean, European and Western Pacific Region. Using the graph builder on JMP the overlay feature compares differences in access to care and treatment by region. The independent variable (access to care) was compared to the x-axis and the dependent variable (WHO regions) to the overlay compartment to get a visual representation of the relationship between WHO regions and access to care and treatment.

## Results

### Demographics

**Fig. 1:**
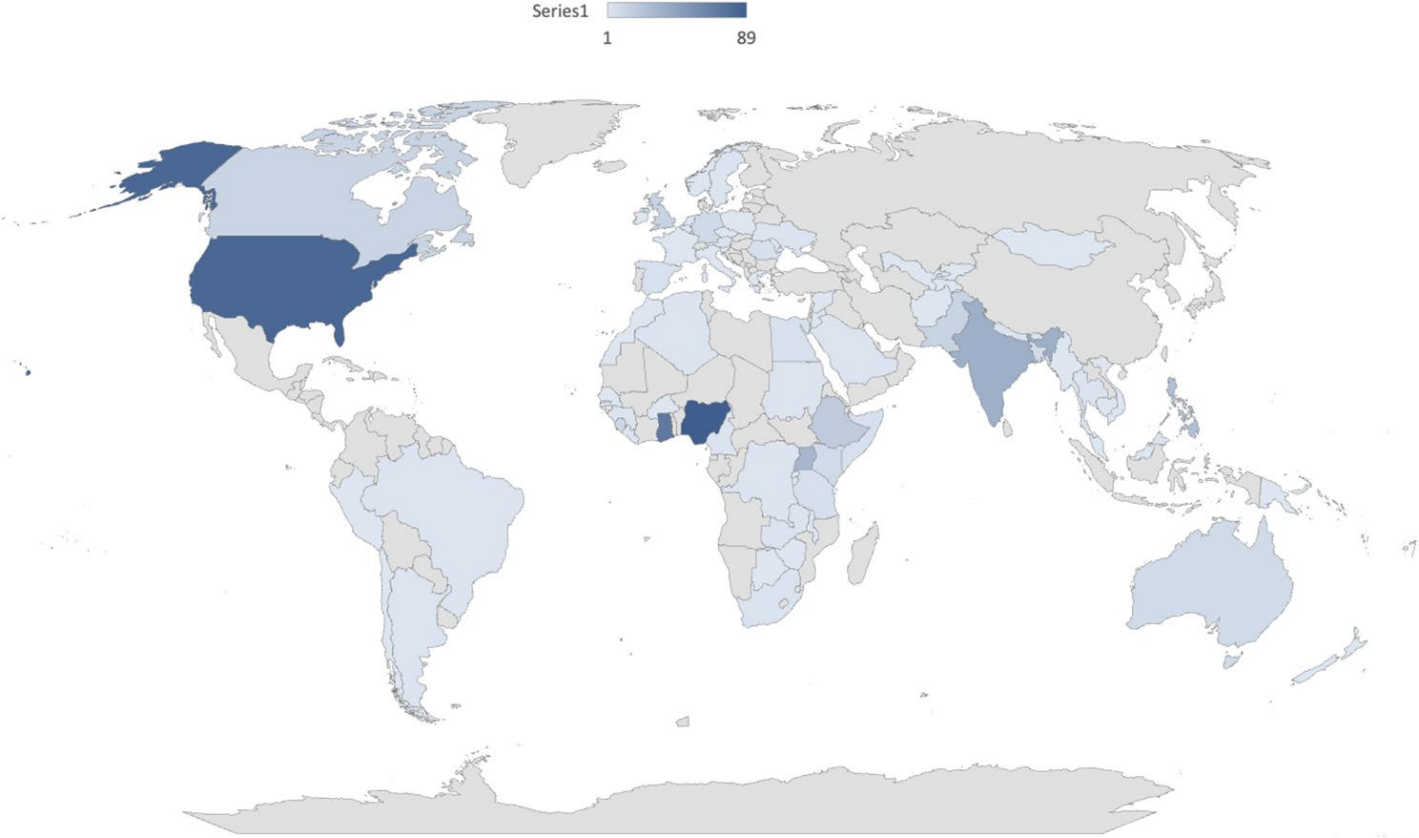
Country of origin as reported from survey participants self-identified as living with hepatitis B (HBsAg positive, *N* = 554)

In total, 755 participants completed the survey; 202 were excluded due to self-reporting not having hepatitis B, leaving a total sample of 560 individuals from 76 countries. The majority of participants reported being from Nigeria (16%, *N* = 91) followed by the United States (15%, *N* = 87), Ghana (13%, *N* = 72), India (7%, *N* = 38), Uganda (6%, *N* = 31), Philippines (5%, *N* = 26), Ethiopia (3%, *N* = 19), United Kingdom (2% *N* = 15), Pakistan (2%, *N* = 13) and Canada (2%, *N* = 12). All other country respondents had less than 2% samples, which can be seen in Fig. 1.

Of the sample, 69% (*N* = 387) identified as male, 63% (*N* = 349) identified as living in an urban area or larger city, while 22% (*N* = 123) lived in a semi-urban or rural area 13% (*N* = 76). Participants’ average age was 39 years old with a range of participants between the ages of 18 to 80 years.

### Hepatitis B medical care

**Fig. 2:**
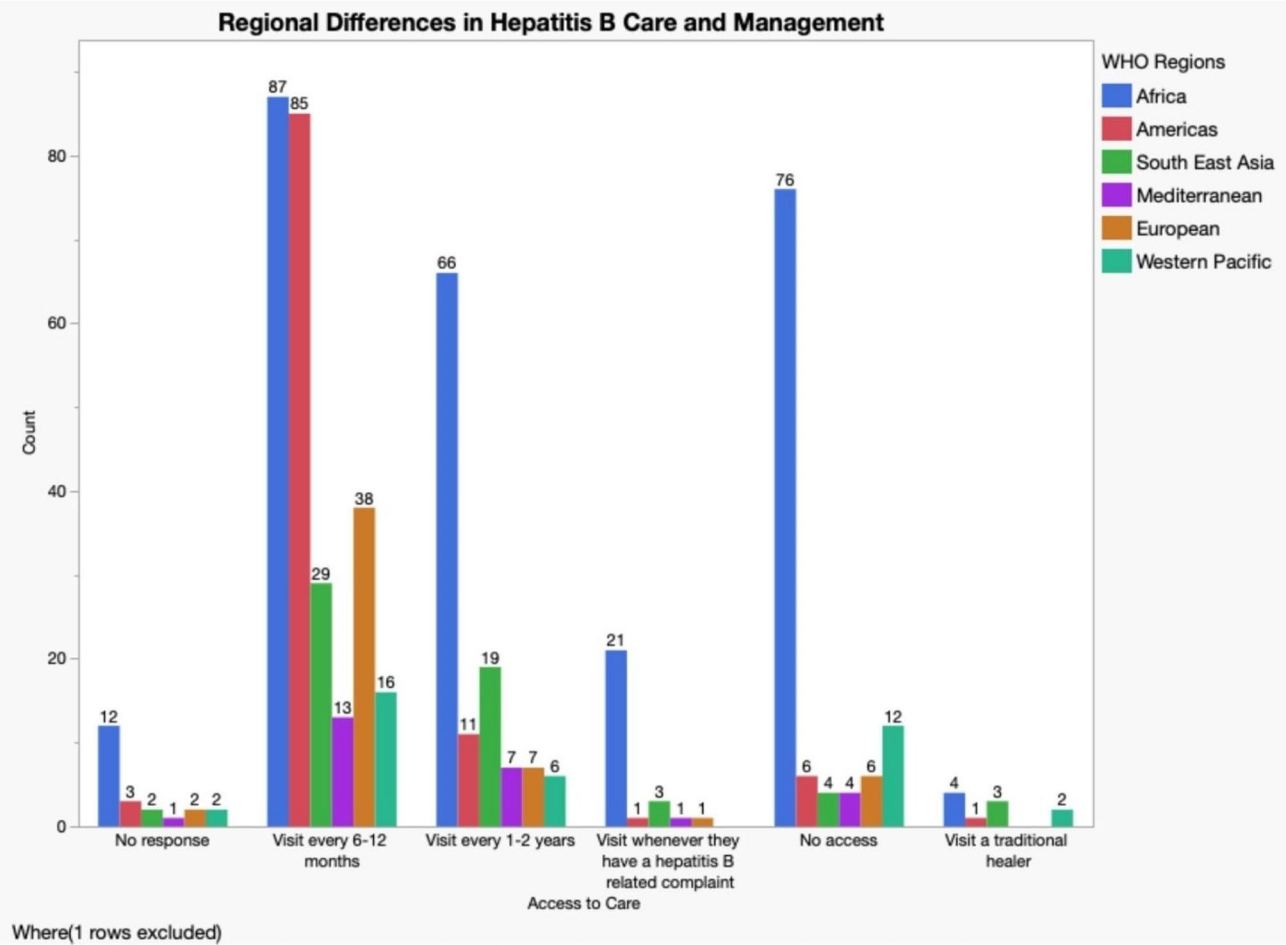
WHO regional differences in access to care based on the WHO hepatitis B management guidelines

**Fig. 3:**
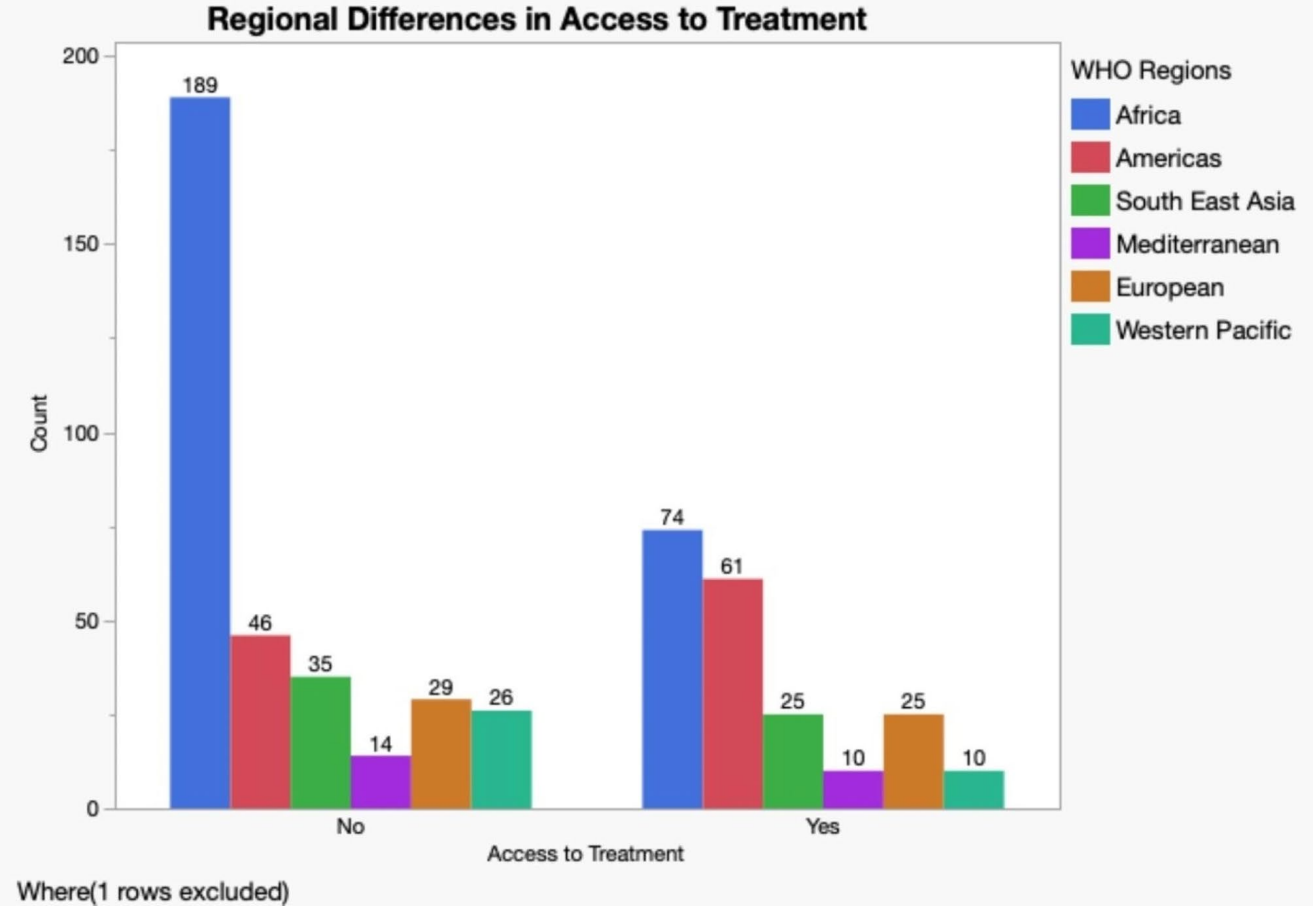
Access to hepatitis B treatment based on WHO region

To identify if the WHO treatment criteria were adhered to and accepted among individuals with hepatitis B, participants were asked to describe the frequency of current health care access for their hepatitis B infection. Less than half (49%, *N* = 268) of respondents reported visiting a doctor to check the health of their liver regularly (every 6–12 months). Others reported visiting the doctor every 1–2 years (22%, *N* = 119), do not have access to a doctor (19%, *N* = 105), or visit the doctor only when they have a new hepatitis B-related complaint (5%, *N* = 28). Several respondents (2%, *N* = 10) reported consulting with a natural healer or herbalist (Table 1). To assess regional differences within access to a provider based on the WHO guidelines individuals were grouped based on country of origin with WHO region (Fig. 2). To help inform the update of the WHO treatment guidelines individuals were asked to participate in an anonymous survey to assess the trajectory of how people with hepatitis B experience and engage with the health care system for their infection from testing, diagnosis, clinical management, treatment and including health care challenges within those settings. Participants were also asked about their specific preferences for overall management and care related to hepatitis B. Respondents reported using antiviral medication, 37% (*N* = 208) with most using tenofovir (69%, *N* = 138) prescribed by a specialist (82%, *N* = 167) or general practitioner (13%, *N* = 26). Individuals were grouped by WHO region and whether or not they were currently on hepatitis B antiviral (Fig. 3). Individuals were asked to report the top reason for starting treatment. Many stated it was recommended by their doctor (30%, *N* = 59); they had a high or elevated hepatitis B DNA/viral load (38%, *N* = 76), or wanted to reduce the risk of getting liver cancer or liver damage (11%, *N* = 23).

**Table 1:** Participants self-reported medical care related to hepatitis B

Which of the following statements best describes your general hepatitis B medical care? (*N* = 548)	Percentage	Count
I visit a doctor regularly (every 6–12 months to check the health of my liver)	49%	4268
I visit a doctor when I can to check the health of my liver (every 1–2 years)	22%	117
I visit the doctor whenever I have a new hepatitis B-related complaint	5%	28
I don’t have access to a doctor that I can visit regularly for my hepatitis B	19%	104
I consult with a natural healer or herbalist	2%	9
Other	2%	10

*Two individuals skipped answering this question

Of people not using treatment, the top reasons were because it was not recommended by their doctor (42%, *N* = 140), or they could not afford the cost of medication (23%, *N* = 77). Others reported feeling healthy so did not feel they needed treatment (11%, *N* = 37), that no one had talked to them about medication (7%, *N* = 22), or they had concerns regarding the treatment side effects (4%, *N* = 14).

### Qualitative responses

Individuals were asked if there was anything they wanted to report on current treatment experiences. A thematic analysis of the open-ended responses (*N* = 114) for this section highlighted major overarching themes including the cost burdens associated with hepatitis B management and treatment. One person from Ghana described, ““*I sometimes skip my medication because it may finish, and I will have to wait for three to six days before I could get it again when I get money*.”” Another individual from Uganda similarly described, ““*It’s quite expensive for both seeing the specialist and buying the drugs*.”” An additional issue was concerns about health workforce skills, with one individual in Zambia described, ““*It seems like most healthcare facilities in Africa, Zambia to be specific are not fully aware of how to treat patients with hepatitis*.””.

### Provider-patient communication & shared decision making

Participants were asked about their current provider-patient interactions and if they had preferences regarding conversations with their providers around treatment initiation. Most participants reported being provided with little to no communication with their provider around reasons to start or not start treatment (Table 2), with half of the participants reporting not being asked by their health providers if they wanted to start treatment (50%, *N* = 256). The overwhelming majority of participants wanted to be involved in the choices around hepatitis B management and treatment (85%, *N* = 435).

**Table 2:** The self-reported provider experiences and preferences related to treatment initiation for hepatitis B

Question	Response Percentage Count		
How much did you and your health care providers talk about the reasons you might want to start hepatitis B treatment? (*N* = 514)	A lot Some A little Not at all	30% 27% 24% 18%	156 141 123 94
How much did you and your health care provider talk about the reasons you might not want to start hepatitis B treatment? (*N* = 509)	A lot Some A little Not at all	17% 21% 25% 38%	84 105 128 192
Did any of your health care providers explain that there were choices in what you could do to treat hepatitis B? (*N* = 513)	Yes No I do not remember	39% 48% 13%	200 247 66
Did any of your health care providers ask if you wanted to start hepatitis B treatment? (*N* = 512)	Yes No I do not remember	44% 50% 6%	227 256 29
Do you want your health care provider to ask you to be involved in choices around your hepatitis B management and treatment? (*N* = 512)	Yes No I have no preference	85% 7% 9%	435 29 48

### Simplified service delivery for testing and treatment

Individuals were asked if they had preferences for hepatitis B testing and management locations, with most preferring hospitals (59%, *N* = 246), followed by laboratories (35%, *N* = 146), family doctors (29%, *N* = 121), or self-testing (20%, *N* = 83). The most important considerations regarding hepatitis B testing were confidence in the doctor’s knowledge of hepatitis B (54%, *N* = 223), followed by testing locations being close to home or work (53%, *N* = 220), the costs associated with the testing or medical fees (42%, *N* = 175), being tested in a non-judgmental atmosphere (25%, N-105), and waiting times (21%, N-90) (Table 3). Most participants supported using telehealth or telemedicine to receive counseling and management for hepatitis B (83%, *N* = 339). The confidentiality of test results was considered important to participants and an important aspect related to receiving a diagnosis of infection (68%, *N* = 280), as was the ability to receive education and counseling for hepatitis B (50%, *N* = 206) by knowledgeable health facility staff (56%, 230), and costs (46%, *N* = 189).

**Table 3:** What are the most important considerations regarding hepatitis B testing locations (*N* = 402)

Answer choices	Percentage	Count
Close to your home or work	53%	220
The doctor’s knowledge of hepatitis B	54%	223
Direct costs (test costs, medical fees)	42%	175
Non-judgmental atmosphere	25%	105
Waiting time	22%	90
Ability to remain anonymous	20%	84
Service hours	16%	66
Presence of community-friendly medical professional	15%	62
Presence of community/peer support	9%	36

When asked after being diagnosed with hepatitis B, when would they prefer to start treatment, one-third of respondents were open to receiving treatment on the same day of a diagnosis (*N* = 30%, *N* = 120), with most willing to start treatment whenever their doctor recommends it (50%, *N* = 202). Rationales for starting treatment on the day of diagnosis were to possibly start treatment more quickly (70%, *N* = 265), reduce possible viral exposure to family and friends (52%, *N* = 191), save time (21%, *N* = 80), and avoid extra costs (21%, *N* = 79).

### Preferences related to hepatitis B testing

When asked about the most important considerations regarding hepatitis B testing many noted the convenience of location, costs, and the doctor’s knowledge as being key aspects of care (Table 2).

### Qualitative responses

Individuals were asked through an open-ended question at the conclusion of the survey if they had any additional thoughts surrounding hepatitis B management or treatment. A total of 317 individuals provided feedback with themes including access to medication, hepatitis B testing, experiences with health care workers, and treatment. Below each theme is described with relevant quotes.

### Access to medication

Within the open-ended responses, 235 individuals responded with comments regarding treatment, with many respondents describing their experiences in accessing treatment in their regions, and their desire for a cure. One individual described the challenges of accessing care in Malawi, *“In Malawi, the public hospitals do not have any formal treatment”*, while another person from Uganda described the importance of accessing care, *“Should consider all people to access and get the best treatment and care without worrying how to afford it.”* Overall, the burden of costs for medication, and testing was described as a significant barrier for individuals an individual from the United Kingdom described, *“mostly in Africa where it is very common, and people can’t afford the cost of testing, medication and lab investigation.”* One person from Ghana noted differential access to treatments across blood borne infections, *“I humbly request that medication for the treatment of Hep[atitis] B should be made easily accessible just like how HIV drugs can be accessed.*”

### Hepatitis B Testing

Respondents also described challenges experienced with hepatitis B testing, with one from Uganda described diagnostic challenges with testing, “*Focusing on improving point of care testing for hepatitis, many patients are lost to care due to difference in time and location of service delivery.”* A person from Ghana highlighted the costs associated with diagnostics being a barrier to care, *“The price for the necessary testing such as Hep B DNA, liver function test, etc. must be affordable to enable Hep B patients [to] get access to treatments.”*

### Hepatitis B Treatment

Respondents described their lived experience in treatment initiation and guidelines, with one person from Ghana noting, *“I think most times, some clients are told per their test results, they don’t need treatment which I was thinking why not start treatment to rather prevent the stage where the case is now serious that, people have to start treatment which sometimes leads to complications.”* One respondent from Pakistan noted, *“We should start treatment as soon as possible everybody”* while one from Sierra Leone shared, *“The timely initiation of medication in my opinion will prevent the condition from getting worse. It is a good way to reduce the spread and to save live[s] and cost[s].”*

One respondent from Nigeria suggested the need for treatment simplification, *“I’d like the treatment guidelines and algorithm to be simplified to test and treat. Also, health insurance cover for people living with hepatitis B should be paramount”*, while another from Uganda was concerned about their pill and treatment burden after being treated for three years, *“If I have been on treatment for over 3 years and my VL (viral load) is non-detected and all other tests and scans are fine - there should be a consideration of stopping treatment-pill burden.”*

The familial impact of hepatitis B infection was described as a motivator with one Gambian respondent sharing, *“I need treatment because my mother died of hepatitis B”*.

One respondent from Nigeria noted a desire for access to treatment after diagnosis, *“I want hepatitis B care to be test[ed] and treat just like HIV/AIDS. Treatment should be commenced immediately irrespective of other hepatitis B serological parameters or liver biochemical parameters.”*

### Health Care workers

From both the quantitative and qualitative responses, the importance of having a knowledgeable doctor or health care provider was significant with one respondent from South Sudan noting the need for a *“knowledgeable health care worker who understands the disease, that I have a doctor that actually cares about me and my hepatitis B.”* Similarly, another individual from Uganda described the importance of provider education and capacity and shared, *“Improve awareness in developing countries, needs to be integrated into other related health services to be offered with no or minimum cost. Peer groups can be supportive to fight stigma and discrimination.”* Another from Nigeria described, *“Health care workers adequately trained on HBV management [are needed].”* Another person from Nigeria further described overarching concerns which highlights the intersection of these themes around care and management and shared, *“Skill and professionalism are required from health workers. Services should be accessible and affordable. The cost of HBV treatment is an inhibiting factor in our case. We are 3 siblings living with HBV and cannot keep up with the costs of routine investigations and treatment and we do not have health insurance.”*

## Discussion

This survey supports previous research identifying barriers to people with hepatitis B accessing care and treatment services and demonstrates insights into the barriers that people with hepatitis B currently experience in accessing standard management and treatment according to the current WHO guidelines. Findings from the data demonstrate a major gap in hepatitis B care and treatment access, particularly from the African region highlighting a major health disparity for people with hepatitis B in a region with high burden.

The 2015 WHO guidelines for hepatitis B recommend regular monitoring to detect liver cancer or HCC (in people with a family history of hepatitis B or cirrhosis) every six months, every 12 months to assess liver enzymes, HBV DNA, and HBeAg, and toxicity monitoring every 12 months.^13^ Most respondents to this survey reported accessing care every one to two years, do not have regular access to a provider or only go when they have a hepatitis B-related complaint. While people responding to this survey are more likely to have better access to health services than most people with hepatitis B in low and middle-income countries, most respondents are not receiving recommended hepatitis B treatment guideline management and results from barriers to accessing care including cost, provider knowledge, and competency managing hepatitis B. Other studies have demonstrated these challenges in accessing recommended care.^6^,^14^–^17^ Interventions should focus on targeting regions where these barriers are most prevalent, particularly within the African context based on findings from this survey.

Overall limited access to care and management can contribute to poorer health outcomes and this has been demonstrated among other health conditions. Participants requested more simplicity and accessibility for their hepatitis B management and treatment, ultimately to reduce barriers to care and treatment including cost. Individuals also highlighted the current WHO recommendation of regular monitoring, which includes HBV DNA testing and routine monitoring of liver enzymes, as limiting the accessibility of standard care. Simplifying guidelines, especially in areas where DNA testing is not available, will improve access to treatment.

There were significant references to how HIV-related care and management were provided, with a desire for hepatitis B care to be provided similarly. One of the hepatitis B antivirals, tenofovir, is used in HIV treatment regimens and PrEP therapy for those uninfected with HIV but at risk for HIV infection due to sexual exposure. Currently, WHO guidelines for HIV recommend antiviral treatment for all people living with HIV including children, adolescents and adults, and pregnant and breastfeeding women, regardless of CD4 cell count. By mid-2018 a total of 163 countries adopted this recommendation which covers 98% of people living with HIV globally.^18^ This treat-all approach to HIV helped lead to substantial gains in antiretroviral coverage for people in sub-Saharan Africa and subsequent improvements in life expectancy and reduced mortality.^19^ Based on our data this concept of a treat-all approach is worth investigating for its acceptability given its potential life-saving implications for people with hepatitis B. Individuals requested to have care more accessible and affordable and the HIV public health-based approach could be a viable option for improving access and simplification of care delivery.

More work needs to be done to ensure shared decision-making as a standard of practice between people with hepatitis B and their health care providers in their management and treatment plans. The vast majority of respondents wanted to be involved in choices in their management but at least half were not asked whether they wanted to start treatment by their providers. It was clear from qualitative responses around treatment that people had various priorities (whether starting treatment immediately to concerns about being on lifelong treatment). For most people with hepatitis B treatment is a lifelong commitment and people with hepatitis B need to be given accurate information to be able to actively participate in shared management decisions.

Respondents wanted knowledgeable health care providers to ensure accurate and appropriate management of their condition with the confidentiality of test results highlighted by those with hepatitis B. This emphasizes the need to ensure the provider workforce is well-equipped with adequate knowledge of hepatitis B management. Additionally, the survey data alludes to the social and psychological consequences associated with a hepatitis B diagnosis like stigma and discrimination.^11^,^20^–^20^ The stigma and discrimination associated with hepatitis B have also been linked to poor knowledge of hepatitis B among both providers and the general public and misconceptions around hepatitis B transmission. 11,21–21 Education surrounding hepatitis B among both the general population and providers is essential to reduce these negative consequences of hepatitis B that impact those diagnosed. The findings highlight the familial nature of hepatitis B infection with family experiences of death and reduced access to treatment impacting families.

If WHO and national hepatitis B elimination goals are to be met by 2030, it is essential that people with hepatitis B actively participate in conversations surrounding guidelines and implementation of care delivery. There are clear gaps in access to care, treatment, and management for hepatitis B described within this survey with a clear desire expressed for the inclusion in medical decisions and improved access to knowledgeable health care services. Future efforts should prioritize ensuring those with lived experiences are part of these global decisions surrounding hepatitis B to ensure their priorities are incorporated and included as we work towards improving access to care and diagnostics and simplification of hepatitis B management.

### Limitations

Our survey cannot represent all the lived experiences associated with hepatitis B and has limitations. First, the survey was only available in the English language using the Survey Monkey platform, limiting the generalizability of its findings to people with English literacy, and it is not accessible within some countries including China, where the global burden of hepatitis B lies. Our study was a convenience sample and recruitment was done through email recruitment within patient advocacy organizations and networks with a subsequent selection bias. People receiving the link for survey participation are likely to have more knowledge associated with hepatitis B compared to others. The survey also uses self-report and self-identification of positive hepatitis B status which adds additional limitations to the study. More than 1/3 of our participants reported being on antiviral therapy, which is higher than most studies, also highlighting possible selection bias as these participants likely are more engaged in care because they are on treatment. Additionally, some individuals did not respond to all questions, though it was minimal it is important to note. While we have a large sample size, there are major global gaps of note, and further efforts are required to capture hepatitis B treatment and management preferences.

#### Ethics approval

**and informed consent to participate.**

Survey participants were informed that the survey was for research purposes with results being used to inform and document the lived experiences of hepatitis B treatment and management as well as preferences for treatment and management globally. Completion of the survey was voluntary and taken as de facto informed consent for participation. Participation in the survey was anonymous, did not collect personal identification data, and was voluntary. The survey and its analysis are ethically reviewed and approved by the Heartland Institutional Review Board (HIRB Project No. 171120-171).

## Data Availability

All data and materials are owned by the authors and can be made available upon reasonable request to the corresponding author Catherine.Freeland@hepb.org.
